# The hidden threat of tobacco use among Chinese adolescents

**DOI:** 10.18332/tid/209425

**Published:** 2025-09-26

**Authors:** Jingru Lin, Chuanwei Ma

**Affiliations:** 1 Beijing University of Chinese Medicine Shenzhen Hospital (Longgang), Shenzhen, China; 2 Department of Epidemiology and Health Statistics, School of Public Health, Guangdong Medical University, Dongguan, China

**Keywords:** adolescents, tobacco, China

**Dear Editor,**

Tobacco use has imposed a substantial disease burden on China^1,2^. Adolescents, undergoing critical physical and mental development, are particularly susceptible to the harms of tobacco use. According to the 2023 China National Youth Tobacco Survey, 4.2% of middle-school students were current cigarette smokers and 2.4% were current electronic cigarette users^3^. These figures highlight the serious threat of tobacco use among adolescents in China, driven by the increasing popularity of flavored e-cigarettes and emerging trends like ‘smoking cards’ that normalize smoking behavior. Flavored e-cigarettes, often marketed with appealing flavors and designs, are particularly attractive to young people. In some regions of China, adolescents can easily purchase e-cigarettes, including flavored ones, as long as they are not wearing school uniforms^4^. The normalization of smoking through games like ‘smoking cards’ further exacerbates the problem, introducing children to smoking culture at an early age.

A survey conducted at a university in Guangzhou, China, found that college students hold a ‘permissive’ attitude toward e-cigarette use: more than 70% reported they would be willing to try an e-cigarette if a friend offered them one^5^. Early exposure to tobacco, whether through traditional cigarettes or e-cigarettes, poses significant health risks. E-cigarettes, despite being marketed as a safer alternative, still contain harmful substances, including nicotine, which can lead to addiction^6^. The normalization of smoking through games and advertisements claiming health benefits misleads adolescents into believing that smoking is socially acceptable. This not only increases the likelihood of smoking initiation but also undermines public health efforts to reduce tobacco use.

To address this issue, comprehensive measures are needed. Strengthening the enforcement of regulations on e-cigarette sales and advertising, increasing public awareness of the health risks of e-cigarettes, and developing targeted interventions to counteract the normalization of smoking are essential^7^. Educational programs that emphasize the dangers of smoking and promote healthy lifestyles can play a vital role in preventing adolescents from starting to smoke^8,9^. The health of future generations depends on our ability to effectively counter these emerging threats and protect young people from the dangers of tobacco use^10^.

## Data Availability

Data sharing is not applicable to this article as no new data were created.
